# Long-term outcomes of argon laser photocoagulation in small size cyclodialysis cleft

**DOI:** 10.1186/s12886-015-0113-0

**Published:** 2015-09-24

**Authors:** Jong Chul Han, Young Kyo Kwun, Seok Ho Cho, Changwon Kee

**Affiliations:** Department of Ophthalmology, Samsung Medical Center, Sungkyunkwan University School of Medicine, Seoul, Republic of Korea

**Keywords:** Argon laser photocoagulation, Cyclodialysis cleft, Outcomes, Prognosis

## Abstract

**Background:**

To evaluate the long-term outcomes of Argon laser photocoagulation compared to surgical direct cyclopexy in small-size cyclodialysis cleft patients.

**Methods:**

This is a retrospective study. Small-size cyclodialysis cleft patients who underwent Argon laser photocoagulation and surgical direct cyclopexy were reviewed. The mean follow-up period were 82.4 (range, 61 – 145) months and 99.9 (range, 62 – 184) months in both groups. The comparison of best corrected visual acuity (BCVA), intraocular pressure (IOP), postoperative peak IOP and time to normalization of IOP before and after the treatment.

**Results:**

The causes of all included 15 cyclodialysis cleft cases were blunt trauma. seven patients underwent Argon laser photocoagulation and eight patients underwent surgical direct cyclopexy. The mean age of included patients was not significantly different (*p* = 0.38). Preoperatively, the mean logMAR BCVA (standard deviation, SD) was 0.7 (0.2) and 1.1 (0.9) and mean IOP was 4.4 (2.4) mmHg and 3.0 (1.5) mmHg in Argon laser group and surgical direct cyclopexy group (*p* = 0.24 and *p* = 0.18, respectively). The extension of cyclodialysis and duration of cyclodialysis cleft were not significantly different between the two groups (*p* = 0.08 and *p* = 0.24, respectively). The mean follow-up period were 82.4 (range, 61 – 145) months and 99.9 (range, 62 – 184) months in both groups (*p* = 0.41). Postoperatively, the mean logMAR BCVA was 0.0 (0.1) and 0.2 (0.3) and mean IOP was 14.5 (3.1) mmHg and 16.8 (2.5) mmHg (*p* = 0.15 and *p* = 0.16, respectively). Postoperative peak IOP and time to normalization of IOP were not different between the two groups (*p* = 0.75 and *p* = 0.91, respectively).

**Discussion:**

It is necessary to use invasive treatment such as cryotherapy or surgical direct cyclopexy in cyclodialysis cleft with hypotonic maculopathy. In the present study, Argon laser photocoagulation showed good prognosis in a small-size cyclodialysis cleft below 1.5 clock-hours. Considering possible complications and cost of surgical direct cyclopexy, Argon laser can be more beneficial than surgical direct cyclopexy in small-size cyclodialysis cleft below 1.5 clock-hours.

**Conclusions:**

The clinical ourcome of Argon laser photocoagulation seems to be as good as surgical direct cyclopexy in small-size cyclodialysis cleft below 1.5 clock-hours.

## Background

Cyclodialysis cleft occurs when the ciliary body is detached from the scleral spur. This is usually related to blunt trauma or iatrogenic injury due to ophthalmic surgery. The communication between the anterior chamber and the suprachoroidal space can result in clinically significant hypotony and its complications such as cataract or macular edema [1]. Clinically, direct suture closure is usually used for repair [2, 3]. However, several alternative methods for closing a cyclodialysis cleft have been used, including penetrating diathermy [4], Argon laser photocoagulation [5], cryotherapy [6, 7], scleral buckling [8], and vitrectomy with gas tamponade [9].

Laser photocoagulation using Argon laser has been used as one of the treatment of choice for repair of cyclodialysis cleft to avoid major intraocular surgeries [10]. Previously, Argon laser photocoagulation in cyclodialysis cleft was introduced by Joondpth [11]. He reported one small-size cyclodialysis cleft case who recovered visual acuity and intraocular pressure (IOP) after Argon laser photocoagulation treatment twice. Harbin also reported that three small-size cyclodialysis cleft patients treated by Argon laser photocoagulation showed good prognosis [12]. However, the follow-up periods of the previous reports were relatively short (2 months to 2 years), there are no long-term outcomes of the Argon laser photocoagulation in small-size cyclodialysis cleft. Therefore, we intended to investigate the long-term outcomes of Argon laser photocoagulation compared to surgical direct cyclopexy in small-size cyclodialysis cleft patients.

## Methods

The present study was retrospective study approved by the Institutional Review Board of Samsung Medical Center and adhered to the tenets of the Declaration of Helsinki. The medical charts of the traumatic cyclodialysis cleft patients who visited Samsung Medical Center and underwent Argon laser photocoagulation from January 1999 to December 2008 were reviewed.

We included the patients with small-size cyclodialysis cleft below the extent of 2 clock-hours. A single surgeon (CK) performed all the Argon laser treatment and surgical direct cyclopexy. All included patients with small-size cyclodialysis cleft used atropine at least for 2 months at first. In case the patients had no improvement of the symptoms of cyclodialysis cleft such as persistent ocular hypotony with decreased visual acuity or macula edema with medical treatment, they underwent Argon laser photocoagulation or surgical direct cyclopexy. The decision which methods the patient would undergo was made by the patient after explaining the strength and weakness of the each treatment. We only included the cases with medical records over five years after the treatment. The cases with severe ocular complications such as scleral laceration and choroidal rupture were excluded. The treatments were fully explained to each patient, and each provided written informed consent.

**Fig. 1:**
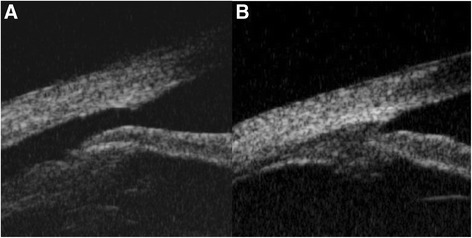
Case A2, A 31-year-old man presented with deteriorated visual acuity of the left eye after blunt trauma. **a** Cyclodialysis cleft was found on preoperative ultrasound biomicroscopy (UBM). **b** Closed cyclodialysis cleft was found on UBM at three months after Argon laser photocoagulation

All patients underwent routine ophthalmologic examinations including best-corrected visual acuity (BCVA), slit-lamp examination, Goldmann applanation tonometry and fundus photo. Gonioscopic examination and ultrasound biomicroscopy (UBM) (Fig. 1a and b) were used in all patients for confirmation of the location and extent of cyclodialysis cleft before laser treatment. After the treatment, the patients underwent ophthalmologic examinations such as BCVA, Goldmann applanation tonometry, gonioscopic examination and fundus examination at 1 week, 1 month and 3 months, and every 6 month. UBM was performed at 3 month after the treatment. Follow-up period was regarded as the duration between initial visit and last visit to outpatient clinic for cyclodialysis cleft. Duration of cyclodialysis cleft before treatment (DOC), postoperative maximum IOP and time to IOP normalization (TNP) were evaluated. Normalized IOP was defined as the IOP between 10 and 21 mmHg.

### Argon laser photocoagulation

The procedure was performed on the outpatient clinic. Retrobulbar anesthesia using 2 % lidocaine was used. If the anterior chamber is too shallow, it was deepend with ophthalmic viscosurgical device (OVD), Hyal 2000® (sodium hyaluronate 1.0 %, LG life sciences, Co., Ltd). The location and extent of cyclodialysis cleft was confirmed by gonioscopy and Argon laser was applied to the full extent of the cleft. The protocol of Argon laser photocoagulation (GYC-1000, Nidek, Co., Ltd) in our clinic was as follows: at first, the laser targeted for bare sclera detached from ciliary body. The recommended spot size was 300 to 500 μm, duration of 0.3 to 0.5 s and the power ranged between 800 and 1000 mW. The green color of laser (532 nm) was used. The number of burns was approximately 40 to 50 shots. And then, the target of laser changed into the area that contains iris root and detached ciliary body. The spot size also changed into 100 μm with a duration of 0.1 to 0.2 s and the power ranged between 600 and 800 mW. The number of burns was approximately 40 to 50 shots. The laser power was titrated based on the tissue response. We regarded bubble formation around tissue as the proper tissue response of an effective laser treatment. If pop sound with bubble formation occurred, the power was decreased. The number of burns was usually totally 80 to 100 shots. After the procedure, patients used 1 % atropine for one month. Because of temporary IOP increase, a topical β-blocker, an oral carbonic anhydrase inhibitor, or an intravenous hyperosmotic agent was used. If the IOP was still low at 1 week, additional Argon laser photocoagulation was performed as the same method mentioned above.

### Surgical direct cyclopexy

A conjunctival flap was made and the sclera was exposed at the site of the cyclodialysis cleft. A partial-thickness scleral flap was made approximately 4 mm posterior to the limbus. After elevating the scleral flap, the sclera was incised 1.5 mm posterior and parallel to the limbus. Aqueous humor was released, and the cyclodialysis cleft was visualized. The detached ciliary body was attached to the sclera with an interrupted suture through the anterior sclera, the ciliary body, and the posterior scleral lip using 10–0 nylon sutures. The scleral flap and conjunctiva were closed. After the surgery, 1 % atropine was used in eyes. Because of temporary IOP increase, a topical β-blocker, an oral carbonic anhydrase inhibitor, or an intravenous hyperosmotic agent was used.

### Statistical analysis

Independent *t*-test or Mann–Whitney *U*-test was used for comparison of the clinical characteristics between Argon laser photocoagulation group and surgical direct cyclopexy group. Clinical characteristics that were compared between the two groups were as follows: Age, extent of cyclodialysis cleft, DOC, preoperative and postoperative final BCVA and IOP, postoperative maximum IOP, TNP, and follow-up period. Statistical analyses were performed using 18.0 (SPSS Inc., Chicago, Illinois, USA). The results were considered significant at *p*-values < 0.05.

## Results

Twenty-one patients with a small-size cyclodialysis cleft were investigated. Five cases were excluded because they were accompanied by ocular problems such as scleral laceration and choroidal rupture. Among 16 patients, one case was recovered from hypotony only using 1 % atropine for a month. Argon laser photocoagulation for cyclodialysis repair was performed in seven eyes of seven patients. Surgical direct cyclopexy were performed in eight eyes of eight patients. The cause of all cyclodialysis cleft cases treated by Argon laser or surgical cyclopexy was blunt trauma. The mean (standard deviation, SD) age of included patients was 33.3 (15.2) years (range, 14 – 50 years) and 40.8 (16.3) years (range, 14 – 60 years) in Argon laser group and surgical direct cyclopexy group (*p* = 0.38). All the included patients were men except one case in surgical direct cyclopexy group.

Preoperatively, the mean (SD) log minimum angle of resolution (logMAR) BCVA were 0.7 (0.2) and 1.1 (0.9) and mean (SD) IOP were 4.4 (2.4) mmHg (range, 1 – 7 mmHg) and 3.0 (1.5) mmHg (range, 0 – 5 mmHg) in both groups (*p* = 0.24 and *p* = 0.18, respectively). The mean extent of cyclodialysis cleft were 1.2 (0.3) clock-hours (range, 1 – 1.5 clock-hours) and 1.6 (0.4) clock-hours (range, 1 – 2 clock-hours) and the duration of cyclodialysis before laser treatment were 6.1 (8.4) months (range, 2 – 25 months) and 2.0 (0.5) months (range, 1 – 3 months) in both groups (*p* = 0.08 and *p* = 0.24, respectively). All patients except one patient (Case A7) had hypotonic maculopathy before the treatment.

Postoperatively, the mean (SD) final logMAR BCVA were 0.0 (0.1) and 0.2 (0.3) and mean (SD) final IOP were 14.5 (3.1) mmHg and 16.8 (2.5) mmHg in both groups (*p* = 0.15 and *p* = 0.16, respectively). The mean (SD) postoperative maximum IOP were 30.4 (13.4) mmHg (range, 15 – 46 mmHg) and 32.8 (13.8) mmHg (range, 17 – 50 mmHg) in both groups (*p* = 0.75). The mean (SD) time to normalization of IOP were 9.6 (15.8) days (range, 1 – 45 days) and 8.9 (6.9) days (range, 1 – 21 days) in both groups (*p* = 0.91). The mean (SD) follow-up period were 82.4 (29.0) months (range, 61 – 145 days) and 99.9 (47.1) months (range, 62 – 184 days) in both groups (*p* = 0.41). There were no complications during the laser treatments or surgeries. One patient (Case A3) in Argon laser group had an additional Argon laser photocoagulation at 1 week after the first Argon laser treatment due to persistent hypotony. Recurrence of hypotony, secondary glaucoma were not found during follow-up period. The preoperative and postoperative demographics of the cyclodialysis cleft patients are summarized in Table 1.

**Table 1:** Preoperative and postoperative demographics

	Patients	Age (years)/Sex	Extent (clock-hour)	DOC (months)	Pre/Post BCVA (logMAR)	Pre/Post IOP (mmHg)	Hypotonic maculopathy	Postmax IOP (mmHg)	TNP (days)	Follow-up (months)
Argon Laser Photocoagulation	Case A1	48/M	1.5	3	0.70/0.00	6/12	+	15	2	68
	Case A2	31/M	1	2	1.00/0.10	7/14	+	46	7	61
	Case A3	48/M	1	2	0.80/0.05	1/11	+	16^a^	2^a^	145
	Case A4	21/M	1	6	0.80/0.05	4/15	+	40	3	75
	Case A5	50/M	1.5	2	0.50/0.00	4/20	+	38	45	90
	Case A6	14/M	1.5	3	0.50/0.00	2/17	+	40	7	68
	Case A7	21/M	1	25	0.30/0.10	7/13	–	18	1	70
	Average (SD)	33.3 (15.2)	1.2 (0.3)	6.1 (8.4)	0.7 (0.2)/0.0 (0.1)	4.4 (2.4)/14.5 (3.1)		30.4 (13.4)	9.6 (15.8)	82.4 (29.0)
Surgical Cyclopexy	Case S1	40/M	2	2	1.40/0.82	3/17	+	42	14	66
	Case S2	49/M	1.5	2	0.52/0.05	5/20	+	45	21	65
	Case S3	30/M	2	2	0.40/0.00	3/15	+	17	3	184
	Case S4	27/F	1.5	2	1.00/0.00	2/18	+	30	3	154
	Case S5	46/M	1	3	0.05/0.00	3/13	+	42	7	120
	Case S6	60/M	1.5	2	1.70/0.52	4/20	+	18	8	76
	Case S7	14/M	1	1	0.70/0.05	0/16	+	18	1	72
	Case S8	60/M	2	2	3.00/0.30	4/15	+	50	14	62
	Average (SD)	40.8 (16.3)	1.6 (0.4)	2.0 (0.5)	1.1 (0.9)/0.2 (0.3)	3.0(1.5)/16.8 (2.5)		32.8 (13.8)	8.9 (6.9)	99.9 (47.1)

No significant differences between the two groups, Independent *t*-test or Mann–Whitney *U*-test

*BCVA* Best corrected visual acuity, *DOC* Duration of cyclodialysis cleft before surgery, *IOP* Intraocular pressure, *SD* Standard deviation, *TNP* Time to normalization of IOP

^a^Measurements after secondary treatment

## Discussion

In the present study, Argon laser photocoagulation showed a good prognosis similar to surgical direct cyclopexy in small-size cyclodialysis cleft below 1.5 clock-hours. No significant differences in preoperative and postoperative clinical characteristics were shown between Argon laser photocoagulation group and surgical direct cyclopexy group. Given that the Argon laser photocoagulation is simpler and less expensive than surgical direct cyclopexy, Argon laser photocoagulation would be a better treatment of choice in small-size cyclodialysis cleft.

When the extent of the cyclodialysis cleft is not large, it is possible to wait for spontaneous recovery using conservative treatment. Previously, several reports suggested that conservative medical treatment was effective in small-size cyclodialysis cleft. Ormerod et al. reported that 1 % atropine can help to close the cleft by relaxation of the ciliary muscle to sclera [13]. Joondeph et al. suggested that abrupt cessation of steroid can make inflammation and help to close the cleft [11]. However, it is difficult to keep observation if the cyclodialysis cleft patients have hypotonic maculopathy that is potentially vision threatening [10]. Visual acuity cannot be recovered due to irreversible wrinkling and fibrosis of the retina [14, 15]. In our case, most of the cases (93.3 %) showed hypotonic maculopathy in preoperative examinations. In addition, it seems not to be effective only using 1 % atropine even in small-size cyclodialysis. In our present study, only one case (6.3 %) was recovered from hypotony by using 1 % atropine only. Therefore, it would be eventually necessary to use invasive treatment such as cryotherapy and surgical direct cyclopexy even though the extent of the cleft is small in cyclodialysis cleft.

Several treatment methods to close cyclodialysis cleft have been introduced before. Surgical direct cyclopexy has been known as the definite treatment of choice in cyclodialysis cleft. However, it is invasive and takes longer and costs more to perform [2, 3]. Transscleral diathermy is also invasive. The surgeon should create a partial thickness scleral flap prior to the application of the treatment and has a risk of inducing thermal tissue damage [4, 16]. Transscleral diode photocoagulation and cryotherapy can be another good treatment of choice. However, the device such as a diode laser or a cryotherapy uncommonly used in the outpatient clinic should be prepared. In this point of view, Argon laser photocoagulation has some benefits compared to other invasive methods. First, it can be performed simply on the outpatient clinic basis. Argon laser is more commonly used in outpatient clinic than other devices mentioned above. Ophthalmologists are usually accustomed to Argon laser because the common laser treatments such as a panretinal photocoagulation or a laser iridotomy are frequently performed on the outpatient clinic basis. In terms of time, it takes shorter to perform than other invasive procedures because it is not necessary to make conjunctival flap or scleral flap. After the anesthesia, the Argon laser photocoagulation can be performed in a few minutes. In addition, it can be performed with securing the view of the cleft directly during the procedure. Therefore, it was possible for clinicians to modulate the power and number of shots based on the tissue reaction.

In previous reports, Argon laser setting was 0.1-0.2 s, 50–200 μm, 400–800 mW and 100 to 200 shots were applied in one session [11, 12]. We used two different settings of Argon laser photocoagulation based on the target tissue response. We regarded bubble formation as the good tissue response to the laser treatment. When the bubble formation occurred, it was usually accompanied by the slight melting changes of the target tissue. We thought the melting changes of the tissues as the sign of inducement of proper inflammation. In our experience, the different settings based on the target tissues seemed good to make proper bubble formation on each target tissues. The reason for this is yet to be certain, but we speculate that the different target tissues with different degrees of pigmentations may be the cause of different requisite laser settings.

In terms of recovery, the two methods showed no clinically different results. Postoperative maximal IOP and time to IOP normalization were not significantly different. Postoperative maximal IOP and the time to IOP normalization are important for clinicians to prevent complications associated with hypertensive phase. Only one case (case A3) in Argon laser group underwent the treatment because of persistent hypotony associated with cyclodialysis cleft. However, cyclodialysis cleft was sealed by additional Argon laser photocoagulation and IOP have been maintained over 10 years.

Surgical treatment can burden the surgeons and patients. Surgical treatment has substantial severe complications such as endophthalmitis or suprachoroidal hemorrhage. Furthermore, the conventional surgical direct cyclopexy takes much longer time to be performed than Argon laser photocoagulation. Even though there was no treatment failure case when using direct cyclopexy in the present study, several reports showed failure cases of direct cyclopexy previously [8, 17]. Therefore it is not certain whether early invasive treatment such as surgical direct cyclopexy is clinically beneficial or not especially when the cleft size is small. As a result, proper interventions to prevent irreversible maculopathy and fibrosis can be delayed. In this point of view, Argon laser photocoagulation can be a good choice for early intervention in small-size cyclodialysis cleft.

## Conclusion

In conclusion, the clinical ourcome of Argon laser photocoagulation seems to be as good as surgical direct cyclopexy. Considering cost, time and possible complications of surgical direct cyclopexy, Argon laser can be more beneficial than surgical direct cyclopexy in small-size cyclodialysis cleft below 1.5 clock-hours.

## Consent

Written informed consent was obtained from the patients (Case A2) for publication.

## Abbreviations

IOP: Intraocular pressure
BCVA: Best corrected visual acuity
UBM: Ultrasound biomicroscopy
DOC: Duration of cyclodialysis cleft
TNP: Time to normalization of IOP
OVD: Ophthalmic viscosurgical device
SD: Standard deviation
